# Air pollution in Delhi, India: It’s status and association with respiratory diseases

**DOI:** 10.1371/journal.pone.0274444

**Published:** 2022-09-20

**Authors:** Abhishek Dutta, Wanida Jinsart

**Affiliations:** Department of Environmental Science, Faculty of Science, Chulalongkorn University, Pathumwan, Bangkok, Thailand; Texas A&M University, UNITED STATES

## Abstract

The policymakers need research studies indicating the role of different pollutants with morbidity for polluted cities to install a strategic air quality management system. This study critically assessed the air pollution of Delhi for 2016–18 to found out the role of air pollutants in respiratory morbidity under the ICD-10, J00-J99. The critical assessment of Delhi air pollution was done using various approaches. The mean PM_2.5_ and PM_10_ concentrations during the measurement period exceeded both national and international standards by a wide margin. Time series charts indicated the interdependence of PM_2.5_ and PM_10_ and connection with hospital visits due to respiratory diseases. Violin plots showed that daily respiratory disease hospital visits increased during the winter and autumn seasons. The winter season was the worst from the city’s air pollution point of view, as revealed by frequency analyses. The single and multi-pollutant GAM models indicated that short-term exposure to PM_10_ and SO_2_ led to increased hospital visits due to respiratory diseases. Per 10 units increase in concentrations of PM_10_ brought the highest increase in hospital visits of 0.21% (RR: 1.00, 95% CI: 1.001, 1.002) at lag0-6 days. This study found the robust effect of SO_2_ persisted in Delhi from lag0 to lag4 days and lag01 to lag06 days for single and cumulative lag day effects, respectively. While every 10 μg m^-3^ increase of SO_2_ concentrations on the same day (lag0) led to 32.59% (RR: 1.33, 95% CI: 1.09, 1.61) rise of hospital visits, the cumulative concentration of lag0-1 led to 37.21% (RR: 1.37, 95% CI:1.11, 1.70) rise in hospital visits which further increased to even 83.33% (RR: 1.83, 95% CI:1.35, 2.49) rise at a lag0-6 cumulative concentration in Delhi. The role of SO_2_ in inducing respiratory diseases is worrying as India is now the largest anthropogenic SO_2_ emitter in the world.

## 1. Introduction

Time and again, the policymakers felt the requirements of understanding the status of air pollution in growing cities and association of short-term air pollution exposures spanning one or a few days on morbidity. This is particularly more relevant for the world’s fast-growing cities to accrue benefits of sustainable development. Epidemiological studies conducted in the past in cities held air pollution responsible for inducing different health hazards. The quasi-poison regression model within over-dispersed Generalized Additive Model (GAM) has been very handy for many researchers for exploring the association of air pollution with different morbidity and mortality [1–6]. In a time series where the respondent variable depends on the nonlinear relationship of independent variables, GAM model finds its best applicability. In GAM, the nonlinear confounders can be controlled using smooth functions to correctly estimate the best connection between dependent and independent variables [7–12]. Accordingly, researchers used the GAM model extensively to indicate the role of air pollution in causing health effects for US and European cities [13, 14].

Chinese and Indian cities frequently grabbed the world’s attention because of increasing air pollution and reported health effects on city dwellers. Indian cities were in the limelight because of the uncontrollable nature of air pollution in already declared polluted cities. Different Chinese cities have been put under strict scanners by the researchers who continuously reported or updated the policymakers on air pollution and health hazards so that policy-level initiatives may defuse the situation. Recently Lu et al. [15] reported that research ably supported the polluted Chinese cities to progress in air pollution control and place the much-needed strategic air quality management system. Another recent article indicated that out of 31 research papers published during 2010–2020 investigating the role of different air pollutants on the health of city dwellers using the GAM model, the majority, i.e., 17 were in the backdrop of Chinese cities and 3 for Indian cities [16]. GAM successfully explored the role of different pollutants in establishing their relationships with different types of respiratory morbidity/mortality for 21 cities of China, India, Iran, Brazil. Denmark and Kuwait (S1 Table). Zhao et al. [17], using GAM, reported that Dongguan city dwellers in China faced the threat of enhanced respiratory diseases due to short term exposure to CO. Song et al. [18] found respiratory diseases amongst the children of Shijiazhuang city of China due to PM_10_, SO_2_, NO_2_ presence in the air. Cai et al. [19], studied the total respiratory diseases mortality of Shenzhen, China, and linked them with PM_2.5_ presence in ambient air through GAM modelling. Liang et al. [20] used GAM model to indicate a direct relationship between pulmonary disease in Beijing with air pollution. Very recently Wang et al. [21] confirmed the role of particulate matter (PM) with pneumonia hospitalizations of children in Hefei, China.

Delhi, the capital city of India, is the second most populated and one of the most polluted cities in the world and should be the obvious choice for pollution and health hazard research. The recent air quality report of IQ Air has ranked Delhi first out of the air-polluted capital cities of 106 countries based on PM_2.5_ concentration [22]. According to WHO, Delhi is the sixth-worst polluted city amongst 13 notable other Indian cities. Indeed, the city-dwellers had terrible times when PM_2.5_ of Delhi stood at 440 μg m^-3^ during October 2019, i.e., 12 times the US recommended level. Past studied blamed the huge transport sector with the largest vehicle stock of the country as the critical emission source [23–27]. Chen et al. [28] demonstrated that local transport emissions and neighboring states contributed dominantly to PM_2.5_ and O_3_ concentration strengthening in Delhi. Sreekanth et al. [29] found high PM_2.5_ pollution persists across all the seasons in Delhi despite pollution control efforts in vogue. In the pan-Indian context, air pollution significantly contributed to morbidity and premature mortality in India for a long time [30]. Sharma et al. [31] reviewed 234 journal papers and noted the knowledge gaps in connecting hospital admissions of patients with air pollution of Delhi. Balyan et al. [32] also noted that a deeper understanding of ambient pollutants at the city level and their effect on morbidity was lacking.

Against the background above, the primary objective of this paper to explore the environmental data of Delhi for confirming the poor air quality status of the city and, after that, assess the role of air pollutants with morbidity (respiratory diseases) through the application of the GAM model. A more profound grasp of the city air quality and influences of ambient air pollution on respiratory diseases is much needed. Such studies may provide all critical information for initiating actions to curb air pollution, health risk, developing public health policies, and above all, a strategic environmental management system for Delhi.

## 2. Study location

As a highly populated and polluted city, Delhi provides an opportunity to apply the GAM model for ascertaining how much the prevailing air pollution is responsible for respiratory diseases of the city dwellers. Delhi has spread over 1,483 km^2^ and a population size of about 11 million per the 2011 census study. With time Delhi emerged as a significant city of the country so far as commerce, industry, medical service, and education are concerned. As per Köppen’s climate classification, Delhi’s climate is extreme with five seasons. The summer is scorching (April–June), while winter is freezing (December-January). The average temperature range during the summer is between 25°C to 45°C, while the winter temperature range is between 22°C to 5°C [33]. The comfortable season spring prevails from February to March, and autumn runs from mid-September to late November. The rainy monsoon season spans almost three months, starting from July. Air pollution varies across seasons due to the influence of climatic conditions [34].

## 3. Materials and methods

### 3.1. Air pollution data

**Fig 1:**
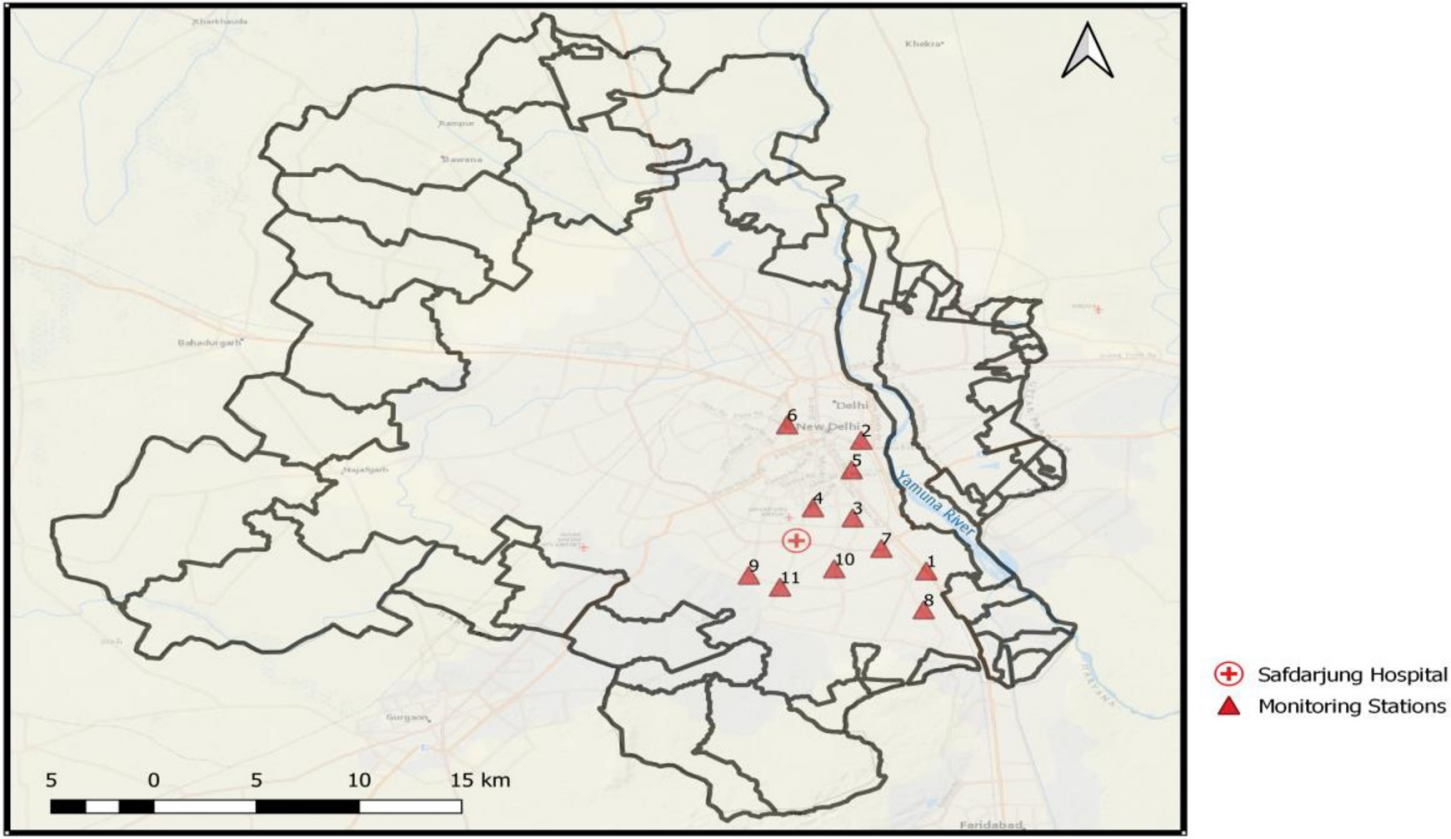
Delhi city, air quality monitoring stations, and hospital location.

Daily average data for three years, January 2016 to December 2018, (1096 data points) of key air pollutants were collected from the State Pollution Control Board (SPCB), Delhi. The pollutants were sulfur dioxide (SO_2_), nitrogen dioxide (NO_2_), carbon monoxide (CO), particulate meter 10 micrometers or less (PM_10_), and particulate meter 2.5 micrometers or less (PM_2.5_) as recorded by 11 NAMP (National Air Quality Monitoring Programme) stations of the city as shown in Fig 1 and S2 Table.

### 3.2. Meteorological data

Time series meteorological data for 1 January 2016–31 December 2018 were collected from Regional Meteorological Department located in Delhi. The data were of a total of 1096 days and included daily average temperature (T), daily average relative humidity (RH), daily average wind speed (WS), and daily rainfall (RF). The collected meteorological and air monitoring data will be adequate to estimate the confounding effect of meteorological conditions on morbidity related to respiratory diseases using GAM model.

### 3.3. Hospital visit data

We considered respiratory diseases covered by J00-J99 under the ICD-10 classification system. Data related to daily hospital outpatient visits of patients for respiratory diseases under International Classification of Diseases-10 (ICD-10), J00-J99 for 2016–2018 (1096 days) were collected from Safdarjung Medical College and Hospital (SMCH) of Delhi. The SMCH had its existence from pre-independence days of India and now functioning under the Ministry of Health and Family Welfare, Government of India. SMCH has many different specialties and super specialty departments, and Respiratory Medicine (RM) is one. Fig 1 shows that all the 11 air pollution monitoring stations considered in this study are located within a road distance of 12 km from SMCH. The hospital records contained information related visit date of patients, age, gender, and final medical diagnosis for each patient. The patient data were grouped age-wise under three categories (i) elderly people (more than or equal to 65 years), (ii) middle-aged (45–64 years), and (iii) young (less than or equal to 44 years). For hospital data collection formal request letter was submitted to the hospital authority. As the data were old data without identifiers and not having any possibility of ascertaining the identities of the individuals to whom the data belong, the hospital waived IRB approval.

### 3.4. Methods of analysis

#### 3.4.1 Summary statistics and analysis of time series

Summary statistics of climatic variables, air pollutants, and hospital visits of the patients such as mean, standard deviation, maximum, minimum, and different quartiles were computed using the SPSS 25 version of the software. Daily hospital visit counts for three years (2016–2018) in SMCH were structured based on the patient’s age, sex, and visit dates. Violin plots were developed for three air pollutants (PM_10_, PM_2.5_, and CO), two climatic variables (T, RH), and hospital visits of patients regarding five seasons of Delhi, indicating the distribution of data prevailing in the city during different seasons. Violin plots have been drawn with XLSTAT statistical software. Time series plots were developed using the SPSS 25 version of the software with time dimensions on the horizontal axis and hospital visits, pollutants and, meteorological variables on the vertical coordinate axes to shed light on the data distribution for three years.

#### 3.4.2 Frequency analysis

The seasonal distribution of PM_2.5_ and PM_10_ concentrations in Delhi during 2016–18 has been done by frequency analysis [35]. Under frequency analysis, first, the city level average concentrations of PM per day were calculated by averaging the concentration of 11 monitoring stations. Then, PM concentrations (both for PM_10_ and PM_2.5_), i.e., number of per day observations for the period 2016–18 falling under six categories like 0–25, 25–50, 50–100, 100–200, 200–300, and more than 300 μg m^-3^ worked out. So, the three-year period (2016–18) data or 1096 observations were segregated session-wise for each of the six categories, and the frequency of their appearances was then expressed in percentage terms. The calculations were done with the help of data analysis ’ToolPak’ of excel. As per the air quality index (AQI) Of India, the range 0–100 is considered a good category, 100–200 as moderate, 200–300 as poor, and above 300 as very poor or severe.

#### 3.4.3 Correlation analysis

To understand the interrelationship between climatic variables and air pollutants data for Delhi (2016–2018), we executed Pearson correlation analysis using SPSS version 25.0 (SPSS Inc., Chicago, IL, USA) software. The coefficients of correlations were established between daily meteorological variables and air pollutants for Delhi. The correlation coefficients at p < .01 were accepted as statistically significant [36]. For better visualization, correlation matrix plots have been drawn with R software’s ’corrplot’ package.

#### 3.4.4 Generalized Additive Models (GAM)

The nonlinear associations of various independent variables (climatic variables and criteria pollutants) and the outcome variable (hospital visits due to respiratory diseases) of Delhi can be better explained by (GAM) model. GAM explicitly allows the relationship between outcome variables independent variables to be developed based on the smooth functions fitted to some independent variables, thereby bringing the flavor of parametric relationships of the covariates in a regression model [37, 38]. Accordingly, in this study, the potential confounding effects of few independent variables that entered the regression model were controlled with non-parametric smoothening splines. Smoothening splines of 7 degrees of freedom (df) per year were fitted to calendar time (time since 1 January 1970) to control long-term trends and possible calendar effects [39, 40]. In line with Wei [41] smoothening splines with 7 df were also applied to mean RH and mean temperature (T) to control their respective confounding effects on the regression model. A linear term of mean wind speed (WS) was allowed to prevail. A dummy variable as the day of the week (DOW) was additionally introduced in the categorical form to control for week effects. As per Peng et al. and Zheng et al. [39, 42], the dfs for smoothing splines were allowed to be determined by the generalized cross-validation (GCV) scores. Finally, based on the description of the regression model formation above, we formed the following GAM model (Eq 1) in our present study with usual notations and applied.

$$Log[E(Y_{i})]=\beta \;x\;X_{i}+s(time,\;df_{1})+s(temperature,\;df_{2})+s(humidity,df_{3})+Wind\;speed+DOW+\alpha$$ (1)

where *i* denotes the day of observation; E (*Y*_*i*_) denotes the daily hospital visits expected due to respiratory diseases; *β* denotes regression coefficient; *X*_*i*_ denotes the daily mean concentration of pollutants; *s* stands for the smoothing spline applied, and *α* is the intercept. Once the basic GAM model is set with the smoothing splines for RH, T, and time variables, the independent variables like PM_2.5_, PM_10_, NO_2_, SO_2_, and CO (per day concentrations) were added to the basic model to make it the multi-pollutant GAM model. We also constructed two single pollutant models for PM_2.5_ and PM_10_, respectively, to understand their respective sole effects on respiratory diseases related to hospital visits in the city under study. In the single-pollutant model, PM_2.5_ and PM_10_ concentrations, in turn, were entered as independent variables in the base model. Generally, single pollutant models do not reflect the synergistic effect of pollutants on morbidity, but in consideration with the multi-pollutant models, they provide crucial complementary understanding.

The respective coefficients of pollutants of the multi-pollutant and single-pollutant GAM models, found out as regression model output, were the inputs in deriving the relative risk (RR) of hospital visits due to one unit rise of each modelled air pollutants in the ambient air.

Past studies have shown that the air pollutants remain in the ambient air and create lingering effects on morbidity. Accordingly, we have considered pollutant concentrations for a single day and multiple days in the study. We tested the lingering effects of air pollution for single-day lags and cumulative lag days. Single-day lag (lag0) means air pollutant concentrations on the same day of the hospital visit, while lag6 indicates air pollutant concentrations of 6 days before the hospital visit. Similarly, for cumulative concentrations of pollutants lag0-1indicate the mean of pollutant concentration of the current day and previous day of the hospital visit (i.e., 2 days mean). Similarly, lag 0–2 indicates the mean of current day pollutant concentration, 1 day before and 2 days before the visit (i.e. 3 days mean). In the same way, lag0-3, lag0-4, lag0-5, and lag0-6 means 4 days, 5 days, 6 days, and 7 days mean pollutant concentrations, respectively. We used single lags of 0, 1, 2, 3, 5, and 5 days (lag0–lag 5) and cumulative lags of 0–1, 0–2, 0–3, 0–4, 0–5, and 0–6 days (lag 0–1 to lag0-6) to explore the lag pattern of health effects in the multi pollutants and single pollutant models. The R software with "mgcv" package (version 4.0.2) was applied to construct the GAM models. For visualizations of GAM models developed in this study, we have used visual tools of the mgcViz R package.

### 3.5. Relative Risk (RR)

Relative risk (RR), often used in epidemiological studies, helps understand the risk of the outcome of an intervened event with non-intervened events. Thus, RR compares one group with another group. In this study, the exposure-response coefficient *β* of pollutants obtained from the GAM models under different lag conditions have been used to estimate RR and their 95% confidence intervals (95% CIs). RR for the *i*^*th*^ predictor variable and its confidence intervals were calculated using the following Eqs 2, 3 and 4.

$$RR_{i}=\exp(\Delta C_{i}\;x\;\beta_{i})$$ (2)

$$CI=\exp\{\Delta C_{i}\;x\;(\beta_{i}-1.96\;x\;S.E_{i})\}\;\text{lower}\;\text{limit}(\text{LL})$$ (3)

$$CI=\exp\{\Delta C_{i}\;x\;(\beta_{i}-1.96\;x\;S.E_{i})\}\;\text{upper}\;\text{limit}(\text{UL})$$ (4)

where Δ*C*_*i*_ is the rise of the *i*^*th*^ pollutant concentration in air and S.E_*i*_ is the standard error of *i*^*th*^ pollutants. Here, Δ*C* will be 1 unit increase in CO and 10 units increase in all other pollutants. RR provides information on the rise of hospital visits due to each unit increase of a pollutant’s concentration level. To make the RR estimates of daily hospital visits due to air pollution more expressive, we also calculated the percentage change (PC, %,) at 95% CI in the following way (Eq 5).

PC = Percentage change of daily hospital visits due to air pollution

$$\text{PC}=(e^{\beta }-1)x\;100\%$$ (5)

In all analyses p-value < 0.05 considered significant.

## 4. Results and discussion

### 4.1 Data distribution and time-series analyses

The distribution of criteria pollutants, climatic variables (T and RH), and daily counts of hospital visits in Delhi are placed in Table 1 for 2016–18. Table 1 indicates that the mean value of PM_2.5_ and PM_10_ concentrations exceeded the guidelines of NAAQS and WHO both by a wide margin. They shoot to as high as 693.08 μg m^-^³ for PM_10_ and 478.25 μg m^-^³ for PM_2.5_ during 2016–2018. The mean RH value of 58.5% (range, 98.3% to 12.5%) in Delhi indicates the city’s humid condition higher than the ideal level relative humidity for health and comfort of 30–50%. The three years mean temperature of 25.63 ± 7.65 °C with a maximum as high as 45°C and a minimum of 0.5°C along with a higher level of RH indicates the extreme climate of Delhi. Daily mean hospital visits of patients for respiratory diseases during 2016–18 was 20±23.52.

**Table 1:** Summary distribution of criteria pollutants, climatic variables, and daily hospital visits (2016 to 2018), Delhi.

Variable	Mean ± SD	Maximum	Minimum	Percentile			IQR
				25th	50th	75th	
Temperature (°C)	25.63 ± 7.65	45.00	0.56	19.44	28.06	31.39	11.94
Relative humidity (%)	58.5 ± 18.76	98.3	12.5	45.98	60.5	72.3	26.33
PM_2.5_ (μg m^-3^)	107.32±71.06	478.25	18.53	54.83	85.93	142.80	87.97
PM_10_ (μg m^-3^)	210.61±95.90	693.08	38.65	140.79	203.28	262.57	121.78
NO_2_ (ppb)	44.60±14.82	101.15	18.13	32.19	43.82	53.93	21.74
SO_2_ (ppb)	14.65±4.35	32.26	6.84	11.43	13.76	16.86	5.43
CO (ppm)	1.40±0.54	5.96	0.54	1.03	1.285	1.62	0.59
Daily Hospital admission	20±23.52	176	0	6	11	28	22

Table 2 reveals that a total of 22,253 patients visited SMCH, Delhi, either for outpatient consultation or admission for respiratory diseases during 2016–2018, as retrieved from hospital records. The maximum number of people who visited the hospital for respiratory ailments for a day was 176, and the minimum 0 patients. Out of the total patients, 63.5% were female, and 30% had ≥65 years of age. Similarly, out of male patients, 52% were aged ≥65 years, as shown in Table 2.

**Table 2:** Gender and age distribution patients of respiratory diseases, Delhi, 2016–2018 (N = 22253).

Variables	Total (N)	Mean	Standard Deviation	Minimum	Maximum
Patients visited	22253	20.30	23.52	0	176
Gender distribution					
Male	8125	7.41	12.04	0	103
Female	14128	12.89	17.37	0	175
Age distribution (Male)					
≥65	4218	72.15	5.15	65	91
45–64	3000	53.23	6.28	45	64
≤44	907	37.61	6.50	4	44
Age female (Female)					
≥65	8009	73.23	6.12	65	93
45–64	5678	56.12	6.30	45	64
≤44	441	37.33	6.23	7	44

**Fig 2:**
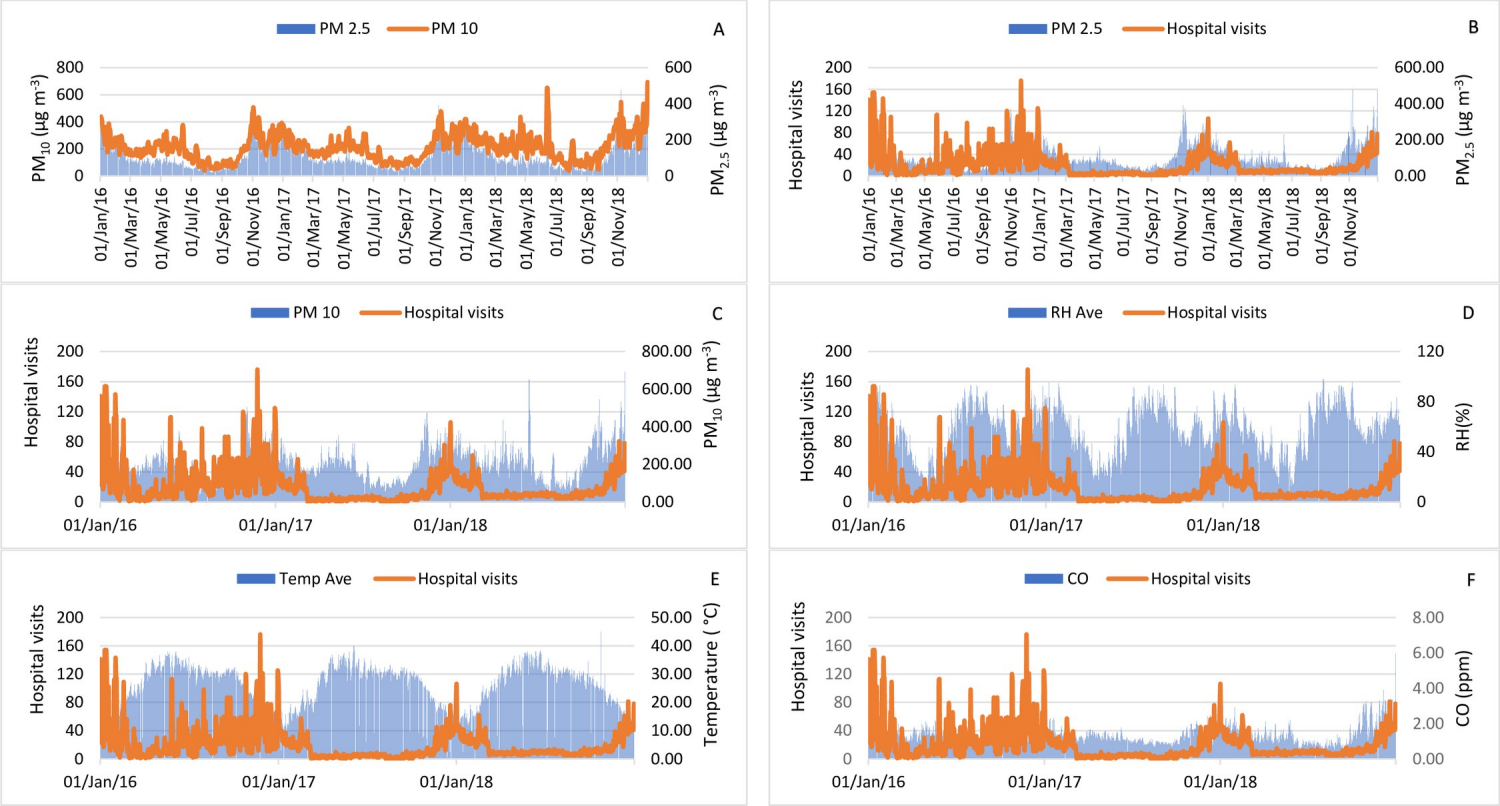
The time series of Delhi from 2016–2018 (A) PM_2.5_ Vs Hospital visit, (B) PM_10_ Vs Hospital visit, (C) RH Vs Hospital visit, (D) T Vs Hospital visit, (E) CO Vs Hospital visit, (F) PM_2.5_ Vs PM_10_.

Time series charts in (Fig 2A–2F) depict behaviors of meteorological variables (RH, temperature), air pollutants (PM_2.5_, PM_10_, and CO), hospital visits, and their interrelationship during 2016–2018 for Delhi. PM_2.5_ and PM_10_ were positively correlated in Delhi during 2016–18, indicating the interdependency (Fig 2A) while maintaining a positive correlation with hospital visits due to respiratory diseases (Fig 2B and 2C). Fig 2D–2E shows that hospital visits tended to negatively correlate with RH and temperature. Fig 2(F) shows a positive correlation of hospital visits with CO concentration too in the city’s environment.

**Fig 3:**
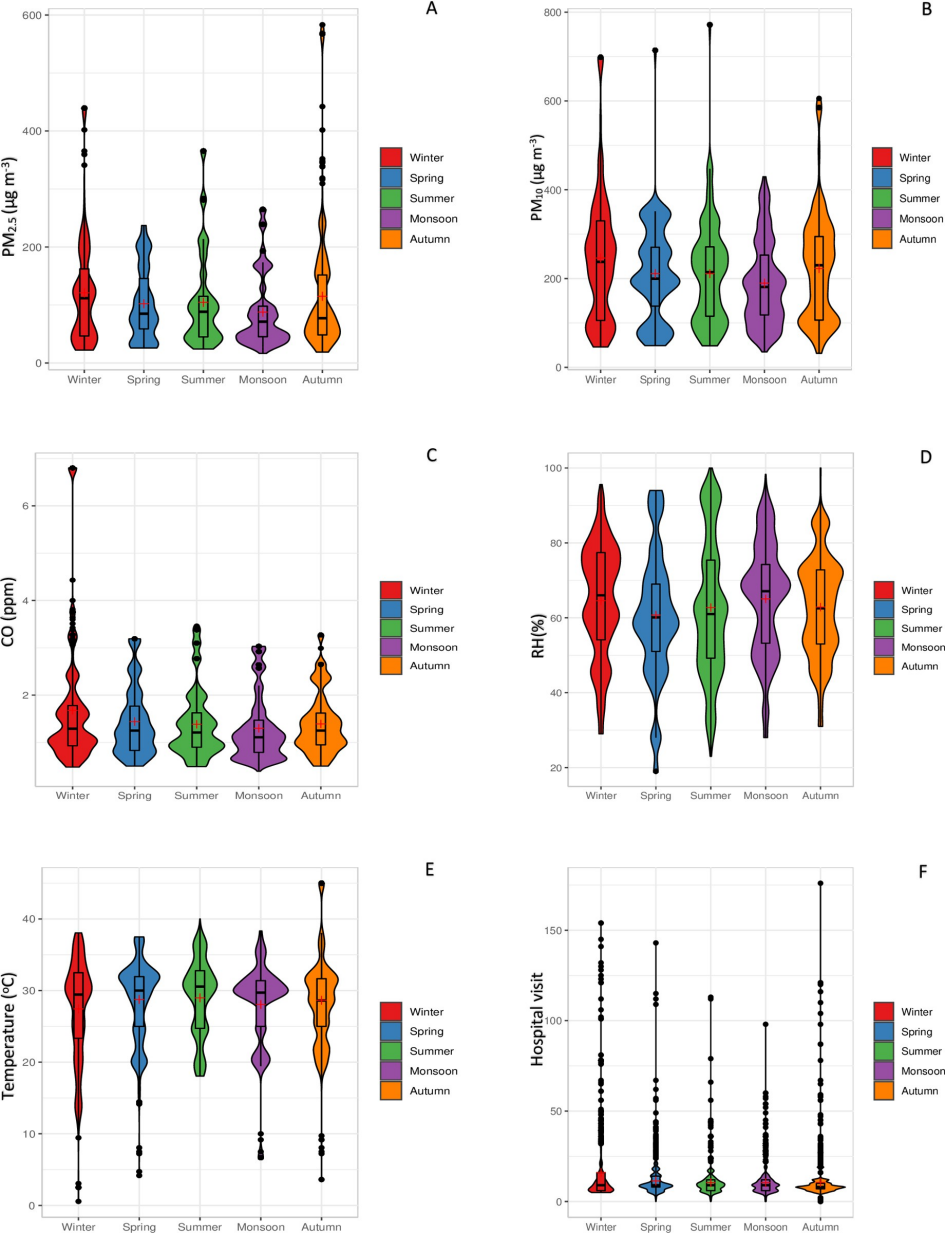
Violin plots of three air pollutants, two metrological variables, and hospital visits in five seasons of Delhi. (A) PM_2.5_, (B) PM_10_, (C) CO, (D) RH, (E) Temperature, (F) Hospital visit.

Violin plots of three air pollutants (PM_10_, PM_2.5_, and CO), two meteorological variables (T, RH), and hospital visits of patients were drawn for the five distinct seasons of Delhi have been provided in (Fig 3A–3F) below. Fig 3A indicates that PM_2.5_ dominates the city environment during winter and autumn. Fig 3B indicates that PM_10_ dominates the city air during the winter and summer seasons, but the median value of PM_10_ concentrations was higher during winter. The concentration of CO in the air remains high during winter and low during the monsoon season (Fig 3C). Fig 3D clearly shows that the city experiences comparatively higher RH during summer and monsoon, with the highest median value during monsoon. Fig 3E indicates that the city experiences the hottest season during summer and autumn. From Fig 3F, it can be observed that during the winter and autumn season’s daily hospital visits due to respiratory diseases increased. The rectangles within the violin plots indicate finishing points of the first and third quartile of data distribution with central dots as medians. The upper and lower whiskers show data spread.

### 4.2 Seasonal distribution of PM_2.5_ and PM_10_ in Delhi

**Fig 4:**
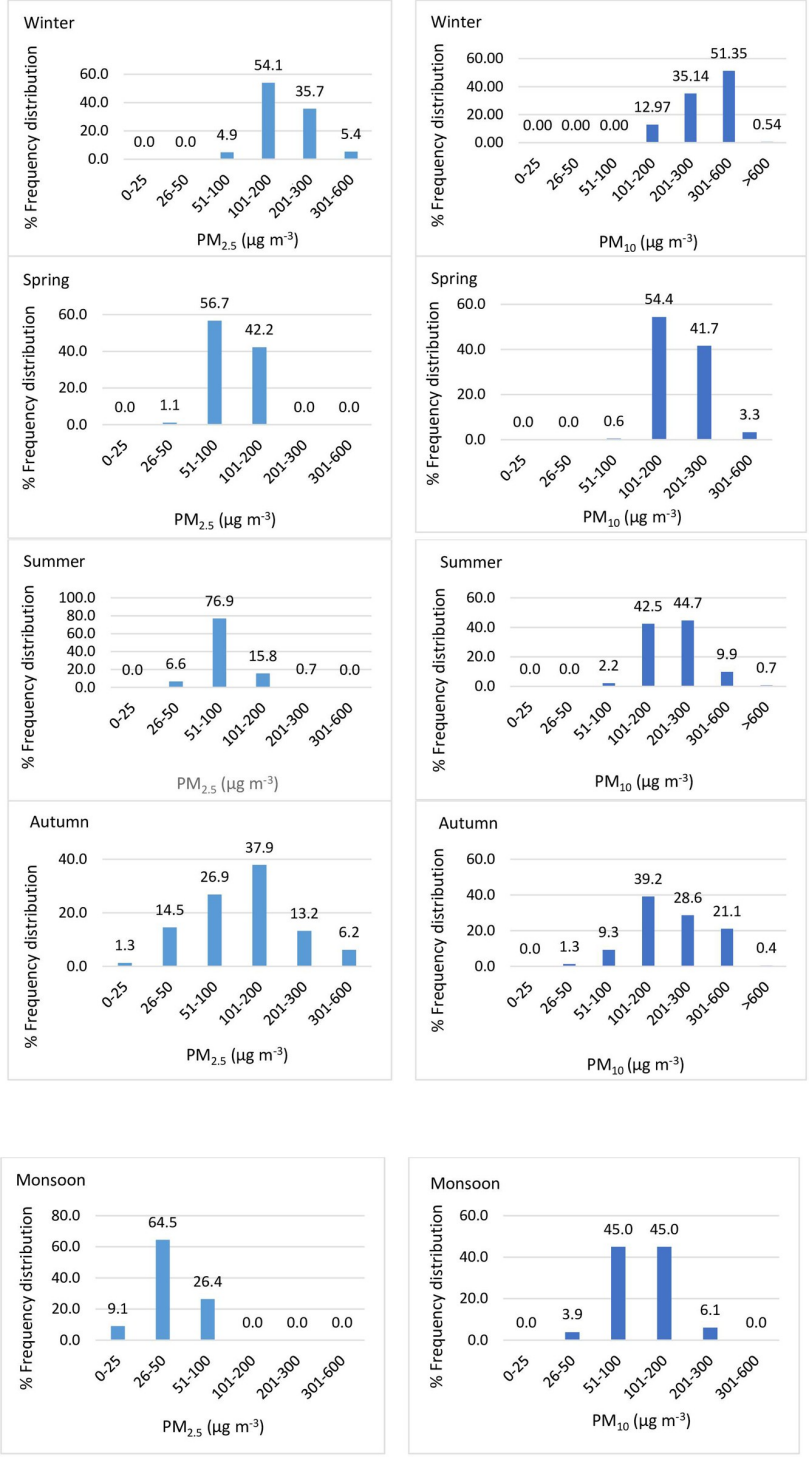
Frequency distribution of PM concentrations across five seasons, Delhi.

The frequency distribution of PM_2.5_ and PM_10_ concentrations for five Delhi seasons are shown in Fig 4. Fig 4 indicates that the winter season was terrible from the air pollution point of view as almost 95.2% of the time, the ambient PM_2.5_ concentrations recorded to be more than 100 μg m^-3^. Alarmingly, 100% of the time, the ambient PM_10_ concentrations crossed the 100 μg m^-3^ benchmark during winter, indicating very harsh wintertime for the city dwellers. The spring season brought some relief for the city dwellers when 42.2% of the time PM_2.5_ concentrations crossed 100 μg m^-3^ benchmark, but PM_10_ remained very strong with 99.4% of the time crossing the 100 μg m^-3^ benchmark. During summer, about 76.9% of the time PM_2.5_ concentrations were under the ’good’ category, and 15.8% of the time PM_2.5_ concentrations were more than the 100 μg m^-3^ benchmark. During summer PM_2.5_ concentrations improved considerably with only 15.8% of the time, its concentrations were more than the 100 μg m^-3^ benchmark, but PM_10_ remained razing with 97.8% time crossing 100 μg m^-3^ benchmark. However, two and half months of monsoon (July, August, and mid-September) brought relief from PM_2.5_ pollution. Almost 100% of the time, PM_2.5_ concentrations remained under the ’good’ category, but PM_10_ remained 51.1% crossing the 100 μg m^-3^ benchmark during monsoon. From autumn (mid-September to late November), PM pollution built up with 97.8% of the time PM_2.5_ concentrations crossing 100 μg m^-3^ benchmark, as shown in Fig 4. In summary, the frequency distribution of PM_2.5_ and PM_10_ concentrations indicates that except winter, the PM concentrations remained very high, which could be a possible cause of health hazards for the city dwellers.

### 4.3 Correlation between pollutants and meteorological variables

**Fig 5:**
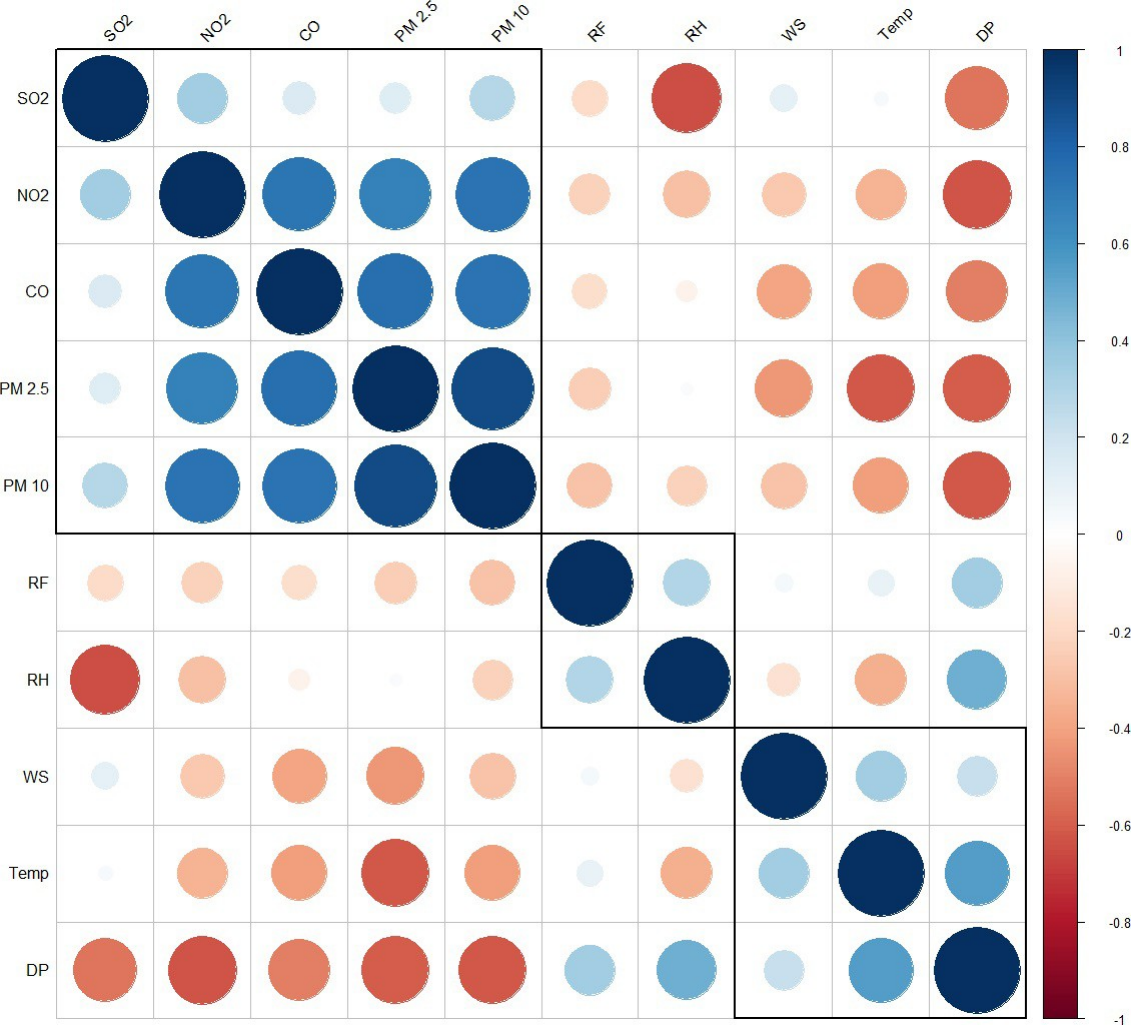
Pearson correlation matrix, 2016–2018, Delhi generated using R program. Blue, red, and while indicate positive, negative, and no correlation respectively.

Positive correlation existed between two important gaseous pollutants SO_2_ and NO_2_ (r = 0.341), while PM_10_ maintained a mild positive correlation with SO_2_ (r = 0.281). PM_10_ almost had linear positive correlation both with NO_2_ (r = 0.783) and CO (r = 0.733) as shown in Table 3 and Fig 5. PM_2.5_ also had positive correlation with SO_2_ (r = 0.137), and positive linear correlation with NO_2_ (r = 0.673) and CO (r = 0.757). Also, PM_10_ and PM_2.5_ maintained positive linear correlation.

**Table 3:** Pearson correlation analysis of variables, 2016–2018, Delhi.

Variables	RF	T	DP	RH	WS	PM_2.5_	NO_2_	SO_2_	CO	PM_10_
RF	1									
T	.097**	1								
DP	.342**	.556**	1							
RH	.299**	-.351**	.482**	1						
WS	0.047	.347**	.220**	-.154**	1					
PM_2.5_	-.240**	-.612**	-.600**	0.024	-.438**	1				
NO_2_	-.227**	-.348**	-.626**	-.299**	-.262**	.673**	1			
SO_2_	-.185**	0.031	-.540**	-.647**	.106**	.137**	.341**	1		
CO	-.173**	-.418**	-.501**	-.064*	-.391**	.757**	.721**	.150**	1	
PM_10_	-.285**	-.412**	-.611**	-.225**	-.289**	.897**	.738**	.281**	.733**	1

**. Correlation is significant at the 0.01 level (2-tailed).

*. Correlation is significant at the 0.05 level (2-tailed).

### 4.4 Association of criteria pollutants with respiratory diseases, Delhi

Multi-pollutant and single pollutant GAM models were formed for Delhi to understand the impact of air pollutants on hospital visits due to respiratory diseases. Multi pollutant models indicate combined effects of the involved pollutants on the hospital visits, whereas single pollutant GAM models cast light on the sole effect of pollutants. The models were tested with different lag concentrations to comprehensively understand the impact of short-term exposure of pollutants on hospital visit counts due to respiratory diseases.

#### 4.4.1. Association of criteria pollutants with respiratory diseases in Delhi (multi-pollutant models)

**Fig 6:**
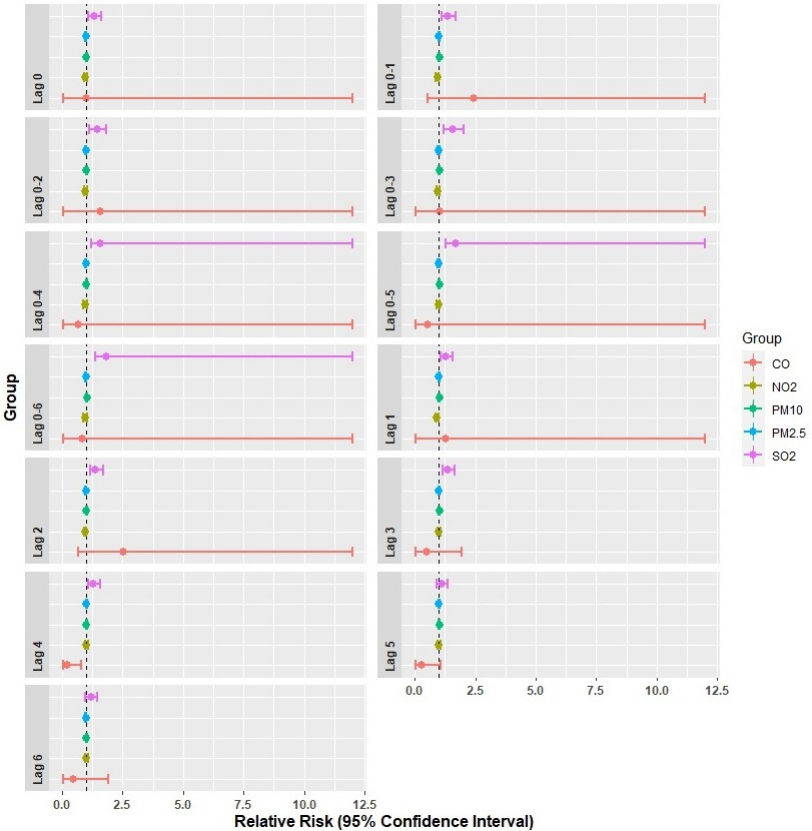
Relative risk pattern (95% CIs) of respiratory diseases related hospital visits in multi-pollutant models, Delhi.

In the multi-pollutant model, criteria pollutants for 2016–18 were included in the base GAM model. Table 4 and Fig 6 indicate the relative risks (RR) of hospital visits due to a rise of 1 unit increase in CO and 10 units for all other pollutant concentrations for different single lag days. The RR patterns in Table 4 indicate synergistic effects of criteria pollutants on respiratory diseases related hospital visits in the city. Table 4 reveals that both PM_2.5_ and PM_10_ concentrations of all the 6 single lag days had no significant effect on respiratory disease-related hospital visits. The effect of NO_2_ on hospital visits was there during lag1 day concentrations only but without any positive acceleration. The effect of SO_2_ on respiratory diseases-related hospital visits was found to be robust instantaneously, i.e., the increase of every 10 ppb SO_2_ on the same day (lag 0) resulted in a 32.6% (RR: 1.326, 95% CI: 1.089, 1.614) rise in hospital visits. The effect of SO_2_ on hospital visits persisted throughout the lag days from lag0 up lag4. The increase in CO on hospital visits throughout the different lag days (lag0 to lag6) was found to be non-significant for respiratory diseases.

**Table 4:** Relative risks of hospital visit due to the rise in concentrations of criteria pollutants in Delhi (multi-pollutant models, single-day lags) *.

	Lag 0			Lag 1		Lag 2		Lag 3		Lag 4		Lag 5		Lag 6	
**PM**_**2.5**_	0.978 (-2.17)			0.993 (-0.71)		0.989 (-1.11)		0.990 (-1.02)		0.993 (-0.66)		0.996 (-0.43)		0.988 (-1.21)	
**CI**	0.957		1.000	0.971	1.015	0.968	1.010	0.968	1.012	0.973	1.014	0.975	1.017	0.967	1.009
**p value**	0.05			0.52		0.31		0.36		0.53		0.70		0.26	
**PM**_**10**_	1.012 (1.21)			1.007 (0.71)		1.007 (0.707)		1.007 (0.736)		1.006 (0.628)		1.008 (0.806)		1.008 (0.847)	
**CI**	0.997	1.027		0.992	1.022	0.992	1.022	0.992	1.023	0.992	1.021	0.993	1.023	0.994	1.023
**p value**	0.12			0.36		0.35		0.34		0.40		0.28		0.26	
**NO**_**2**_	0.958 (-4.213)			0.920 (-8.045)		0.946 (-5.401)		0.993 (-0.660)		1.007 (0.696)		0.986 (-1.386)		1.004 (0.429)	
**CI**	0.904	1.015		0.866	0.977	0.892	1.003	0.937	1.054	0.949	1.069	0.929	1.047	0.946	1.067
**p value**	0.15			<0.001		0.63		0.83		0.82		0.65		0.89	
**SO**_**2**_	1.326 (32.60)			1.278 (27.77)		1.384 (38.42)		1.371 (37.14)		1.296 (29.57)		1.119 (11.87)		1.180 (18.03)	
**CI**	1.089	1.614		1.048	1.558	1.142	1.677	1.130	1.665	1.069	1.571	0.919	1.362	0.970	1.437
**p value**	<0.001			< 0.01		<0.01		<0.01		<0.01		0.26		0.10	
**CO**	0.971 (-2.85)			1.286 (28.58)		2.521 (152.13)		0.493 (-50.73)		0.191 (-80.93)		0.267 (-73.31)		0.467 (-53.25)	
**CI**	0.273	3.456		0.318	5.200	0.657	9.680	0.125	1.947	0.047	0.779	0.066	1.079	0.114	1.923
**p value**	0.96			0.72		0.18		0.31		0.02		0.06		0.29	

*Figs. in the brackets indicates PC (% change in hospital visits)

Note: p < 0.05, p < 0.01, and p < 0.001 considered significant

Table 5 and Fig 6 below indicate the relative risks (RR) pattern of change in hospital visits due to a rise of 1 unit increase in CO and 10 units for all other pollutant concentrations for different cumulative concentrations of pollutants. Both for PM_2.5_ and PM_10_, in terms of cumulative days effect of air pollution, no significant effect could be found. NO_2_ and CO were also not significantly responsible for enhancing respiratory diseases in the city. However, per 10 ppb rise in cumulative lag days, concentrations of SO_2_ led to a comparatively more robust effect on respiratory diseases than single-day lag effects. At lag0-1 per 10 ppb, rise in concentrations of SO_2_ was associated with the percentage change in hospital visits of 37.21% (RR: 1.372, 95% CI: 1.107, 1.701), which increased to 83.34% (RR: 1.833, 95% CI: 1.351, 2.489) during the lag0-6 day. The result indicates the robust effect of pollutants SO_2_ on respiratory disease-related hospital visits in Delhi.

**Table 5:** Relative risks of hospital visit due to the rise in concentrations of criteria pollutants in Delhi (multi-pollutant models, cumulative lag days) *.

	Lag 0–1		Lag 0–2		Lag 0–3		Lag 0–4		Lag 0–5		Lag 0–6	
**PM**_**2.5**_	0.986 (-1.44)		0.981 (-1.93)		0.984 (-1.59)		0.984 (-1.56)		0.978 (-2.16)		0.976 (-2.36)	
**CI**	0.963	1.009	0.957	1.005	0.958	1.011	0.957	1.012	0.951	1.007	0.947	1.006
**p value**	0.22		0.12		0.24		0.27		0.14		0.12	
**PM**_**10**_	1.009 (0.92)		1.010 (1.03)		1.010 (0.96)		1.012 (1.24)		1.016 (1.61)		1.017 (1.67)	
**CI**	0.993	1.025	0.993	1.028	0.991	1.028	0.993	1.032	0.996	1.037	0.995	1.039
**p value**	0.25		0.23		0.30		0.21		0.13		0.14	
**NO**_**2**_	0.928 (-7.20)		0.955 (-4.52)		0.960 (-3.95)		0.956 (-4.40)		0.962 (-3.82)		0.963 (-3.74)	
**CI**	0.871	0.989	0.893	1.021	0.895	1.031	0.888	1.030	0.890	1.040	0.887	1.045
**p value**	0.02		0.18		0.27		0.23		0.33		0.36	
**SO**_**2**_	1.372 (37.21)		1.429 (42.87)		1.553 (55.27)		1.573 (57.34)		1.684 (68.44)		1.833 (83.34)	
**CI**	1.107	1.701	1.130	1.806	1.206	1.999	1.201	2.061	1.262	2.248	1.351	2.489
**p value**	<0.01		<0.01		<0.01		<0.01		<0.01		<0.01	
**CO**	2.447 (144.68)		1.577 (57.68)		1.038 (3.79)		0.671 (-2.86)		0.543 (-5.70)		0.814 (-8.61)	
**CI**	0.541	11.068	0.296	8.413	0.168	6.415	0.094	4.787	0.064	4.603	0.081	8.197
**p value**	0.25		0.59		0.97		0.69		0.58		0.86	

*Figs. in the brackets indicates PC (% change in hospital visits)

Note: p < 0.05, p < 0.01, and p < 0.001 considered significant.

**Fig 7:**
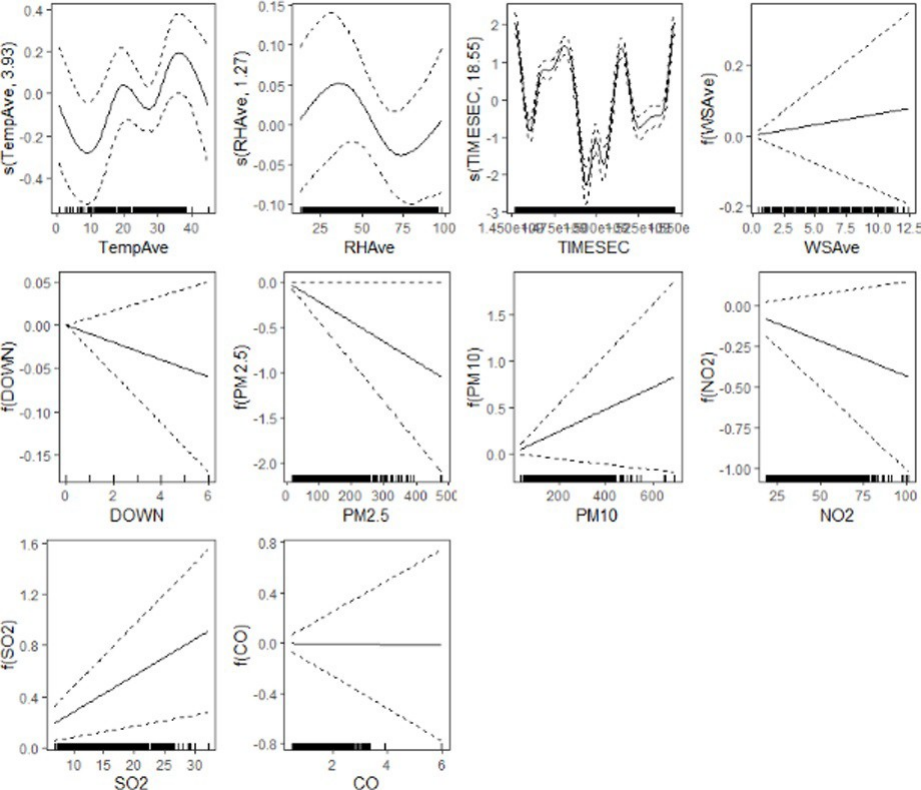
Exploratory variables with confidence bands and smoothers for Delhi city.

**Fig 8:**
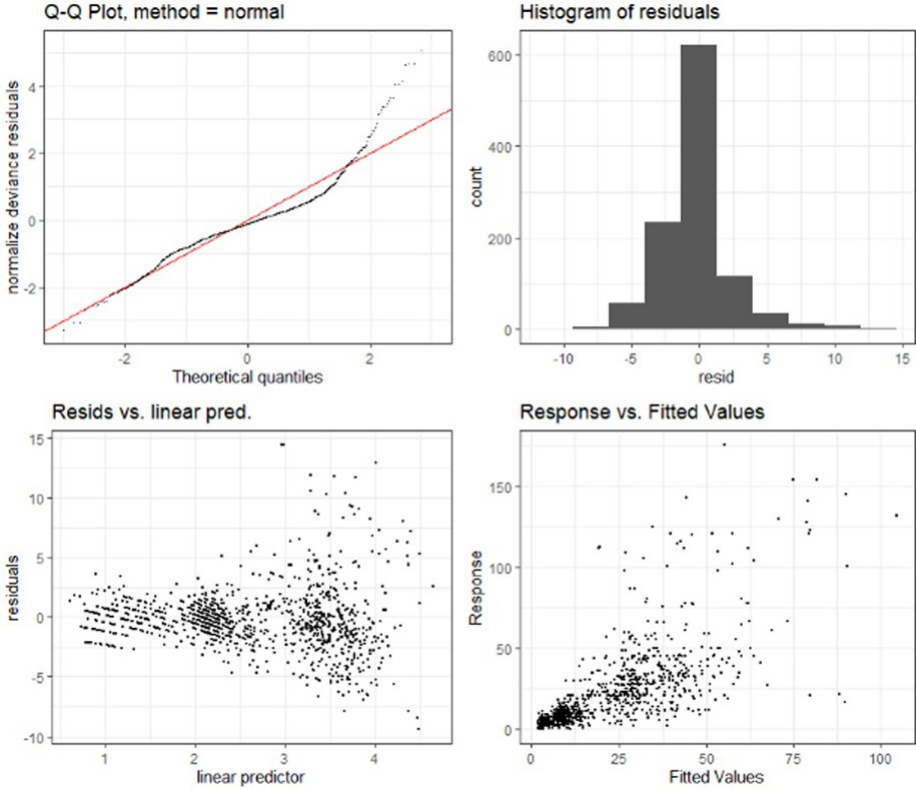
GAM model performance for Delhi city.

Figs 7 and 8 below, drawn with the "mgcViz" R software package (Fasiolo et al., [43], provide the visual representation of the smoothing applied to the non-parametric terms and performance of the GAM model at lag0 respectively.

#### 4.4.2. Association of criteria pollutants with respiratory diseases in Delhi (Single-pollutant models)

**Fig 9:**
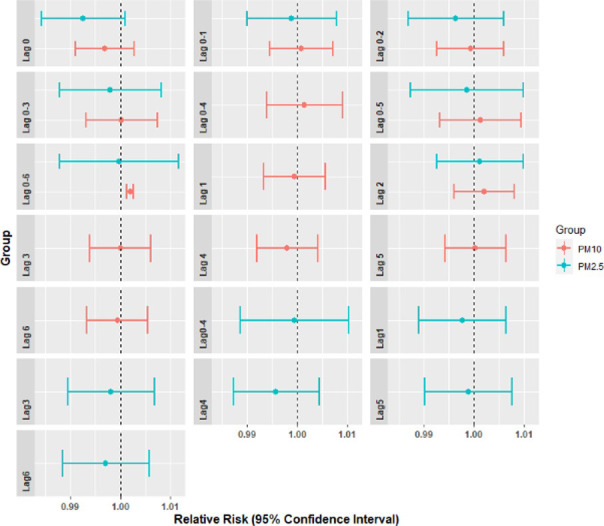
Relative risk pattern (95% CIs) of respiratory diseases related hospital visits in single pollutant models, Delhi.

Two single-pollutant models were developed with pollutants PM_2.5_ and PM_10,_ respectively, to understand the sole effect of PM pollution on respiratory diseases. We fitted different single lag days and cumulative lag days to express the association of daily hospital visits for respiratory diseases with a 10μg m^-3^ increase in PM_10_ or PM_2.5_ in Delhi. Both PM_2.5_ and PM_10_ did not show any significant association with the number of respiratory disease-related hospital visits in Delhi for all the single lag days considered here, as revealed by the p values (Table 6 and Fig 9). In other words, the association of PM_2.5_ and PM_10_ with the respiratory disease was negligible as RR was found to be less than the baseline (RR<1).

**Table 6:** Relative risks of hospital visit due to rise in concentrations of PM (PM_2.5_ and PM_10_) pollutants in Delhi, India (single-pollutant models) *.

Lag days	Pollutants and RR		PC (%)	LL	UL	p value	Lag days	Pollutants and RR		PC (%)	LL	UL	p value
Lag 0	PM_2.5_	0.993	-0.74	0.984	1.001	0.08	Lag 0–1	PM_2.5_	0.999	-0.11	0.990	1.008	0.81
	PM_10_	0.997	-0.31	0.991	1.003	0.30		PM_10_	1.001	0.08	0.995	1.007	<0.05
Lag1	PM_2.5_	0.998	-0.24	0.989	1.006	0.60	Lag 0–2	PM_2.5_	0.996	-0.36	0.987	1.006	0.46
	PM_10_	0.999	-0.05	0.993	1.006	0.86		PM_10_	0.999	-0.07	0.993	1.006	0.84
Lag 2	PM_2.5_	1.001	0.12	0.993	1.010	0.79	Lag 0–3	PM_2.5_	0.998	-0.21	0.988	1.008	0.69
	PM_10_	1.002	0.21	0.996	1.008	0.50		PM_10_	1.000	0.02	0.993	1.007	<0.01
Lag 3	PM_2.5_	0.998	-0.19	0.989	1.007	0.67	Lag 0–4	PM_2.5_	0.999	-0.06	0.989	1.010	0.92
	PM_10_	1.000	0.00	0.994	1.006	0.99		PM_10_	1.001	0.15	0.994	1.009	<0.01
Lag 4	PM_2.5_	0.996	-0.42	0.987	1.004	0.34	Lag 0–5	PM_2.5_	0.999	-0.14	0.987	1.010	0.80
	PM_10_	0.998	-0.20	0.992	1.004	0.53		PM_10_	1.001	0.12	0.993	1.009	<0.05
Lag 5	PM_2.5_	0.999	-0.12	0.990	1.008	0.79	Lag 0–6	PM_2.5_	1.000	-0.03	0.988	1.012	0.96
	PM_10_	1.000	0.03	0.994	1.006	0.93		PM_10_	1.002	0.21	1.001	1.003	<0.05
Lag 6	PM_2.5_	0.997	-0.29	0.989	1.006	0.51							
	PM_10_	0.999	-0.06	0.993	1.005	0.84							

*Note: p < 0.05, p < 0.01, and p < 0.001 considered significant

However, in cumulative exposure single-pollutant models, PM_10_ was found to have persistently enhanced hospital visits of patients with the respiratory disease excepting lag 0–2 days, as shown in Table 6. Table 6 shows that per 10 units increase in concentrations of PM_10_ brought the highest increase in hospital visits of 0.21% (RR: 1.002, 95% CI: 1.001, 1.002) at lag0-6 days. PM_2.5_ association with respiratory disease-related hospital visits found to be non-significant during all the cumulative lag days considered.

## 5. Conclusion and discussion

The study investigated first the level of air pollution in Delhi and then assessed the impact of air pollution on respiratory diseases. The result suggests that Delhi has been struggling to cope up with the increasing nature of criteria pollutants in the first place. A total of 22,253 patients visited the Delhi hospital either for outpatient consultation or admission for respiratory diseases for 2016–2018. The study found that the mean value of PM_2.5_ and PM_10_ concentrations for the period 2016–2018 were 107.32±71.06 μg m^-3^ and 210.61±95.90 μg m^-3^ for Delhi, respectively, which were substantially higher than the NAAQS and WHO standards. Out of the five seasons in Delhi, the winter season is hugely dominated by PM_2.5_ and PM_10_ pollution, as revealed by frequency analyses. Initial time series analysis revealed that PM_2.5_ maintained a positive correlation with PM_10_ have while PM_2.5_, PM_10_, and CO maintained a positive correlation with hospital visits during 2016–18 in Delhi. Pearson correlation analysis confirmed that PM_10_ in Delhi had almost positive linear correlations with NO_2_ and CO while PM_10_ maintained a strong positive correlation with PM_2.5_. Interestingly, SO_2_ too maintained a significant positive correlation with PM_2.5_, PM_10_, NO_2_, and CO. Previous studies in the Indian city of Mumbai highlighted the strong positive correlation of PM_2.5_ with NO_2_ and referred to them as a dummy indicator of air pollution due to transport-related emissions in the city [44]. In the same line, significant positive correlations between PM concentrations and gaseous pollutants, shown by air pollution data, point towards transport-related pollution, solvent evaporation, and waste disposal as sources [45, 46].

This study shows PM_10_ to have persistent enhancing effects on the number of hospital visits with the respiratory disease during all the cumulative lag days excepting lag 0–2 days. Luong et al. [47] reported PM_10_ and respiratory disease-related hospital admission in polluted Hanoi city of Vietnam. Past studies confirmed the role of PM in inducing oxidative stress in the human respiratory system [48]. PM_10_ impact on respiratory diseases in Delhi may be aggravated due to the road dust fraction of PM_10_ that has significant oxidative potential [49]. It was interesting to note that in multi-pollutant models, the role of PM_10_ causing respiratory diseases got subdued due to the combined presence of other pollutants in Delhi city.

This study found that short-term exposure to SO_2_ and PM_10_ led to increased hospital visits of the city dwellers due to respiratory diseases under (ICD-10) J00-J99. The present study reports the mean SO_2_ in ambient air for three years (2016–18) as 14.65 ppb or 38.25 μg m^-3^. SO_2_ is a very critical gaseous pollutant connected with public health [50]. Past studies reported that an ordinary person could withstand only 2.62 μg m^-3^ of SO_2_ in the ambient air without any respiratory problem [51]. However, short but higher concentration exposure to SO_2_ gas can cause persistent pulmonary problems [52]. Orellano et al. [53], in a more recent and extensive review and metadata analysis, confirmed that short-term exposure to SO_2_, varying from few hours to days, can lead to an increased risk of respiratory morbidity/mortality. Our findings agree with that and found a robust effect of SO_2_ on respiratory diseases hospital visits in Delhi. This study shows the robust effect of SO_2_ persisted in Delhi throughout the single lag days (from lag0 up lag4) and had an instantaneous (same day, lag 0) increase of 32.6% (RR: 1.326, 95% CI: 1.089, 1.614) of hospital visits. The cumulative concentrations of SO_2_ were more robust than the single lag day concentration in Delhi. While every 10 μg m^-3^ SO_2_ concentrations on the same day (lag0) showing 32.59% (RR: 1.326, 95% CI: 1.089, 1.614) rise of hospital visits, the cumulative concentration on the day and its previous day (lag0-1) showing 37.21% (RR: 1.372, 95% CI: 1.107, 1.701) rise in hospital visits which further increased to even 83.33% (RR: 1.833, 95% CI: 1.351, 2.489) rise at a lag0-6 cumulative concentration of the pollutant in Delhi. Ren et al. [54], using the GAM model, confirmed the SO_2_ effect on respiratory diseases in the fast-industrializing Chinese city of Wuhan and found that a 10 μg m^-3^ rise in SO_2_ concentrations led to a rise of RR for respiratory disease mortality by 1.9% at lag0 day or same day. More recently, another two highly industrializing cities of Zhoushan and Hangzhou of China with the comparatively lesser presence of average SO_2_ of 6.12 μg m^-3^ and 17.25 μg m^-3^ in ambient air, respectively, confirmed the active role of SO_2_ in enhancing hospital visits of the patient for respiratory diseases [55]. Phosri et al. [56] also reported the effect of SO_2_ for hospital admissions for respiratory diseases in industrializing Bangkok city of Thailand.

Recent COVID-19 and air pollution studies in Delhi indicated that even during the rigorous ’lockdown’ period, there was only a marginal decrease of mean SO_2_ in the ambient air than in the regular times [33, 57]. Therefore, it proves that a significant portion of ambient SO_2_ in Delhi is likely to be from non-local origins like distant transfer, fossil fuel-fired thermal power plants in the bordering areas of Delhi, and biomass burning in the neighboring states. India’s recognition as the largest anthropogenic SO_2_ emitter replacing China in recent times will be much more worrisome in the context of this study’s findings [58, 59].

Suneja et al. [60], through an experimental study in Delhi, reported the seven-year (2011–2018) mean value of SO_2_ level was 2.26 ppb, while this study found a much higher three-year average (2016–18) of 14.65 ppb, indicating the rise of SO concentrations in Delhi in the more recent years. The association of respiratory diseases with PM_10_ and SO_2_ was found stable in different lag days analyses, indicating the problem’s depth for the city dwellers. The robust and instantaneous nature of the relationship between SO_2_ and respiratory morbidity indicated in this study and evidence of similar relationships found in the previous studies highlight the necessity of taking policy-level measures to reduce SO_2_ in the ambient air. Limited GAM model application in Indian cities to link air pollution and health effects is not a limitation of the present study findings but rather a call for more sponsored research in the area.

## Supporting information

S1 Table Air pollutants and their association with respiratory mortality/morbidity for 18 cities using GAM model during 2000–2020. (DOCX)

S2 Table Monitoring stations and their geographic coordinates, Delhi. (DOCX)
