# Supplementary material for: Air pollution in Delhi, India: It’s status and association with respiratory diseases
Source: PLoS One. 2022 Sep 20;17(9):e0274444. doi: 10.1371/journal.pone.0274444 (PMC9488831; doi:10.1371/journal.pone.0274444)
Supplement: S1 Table — (DOCX) [file pone.0274444.s001.docx]

**Air pollution in Delhi, India:** **Its status and association with respiratory diseases**

Abhishek Dutta ^1, *^, Wanida Jinsart ^1^

^a^ Department of Environmental Science, Faculty of Science, Chulalongkorn University, 254 Phayathai Road, Pathumwan, Bangkok 10330, Thailand

^*^ Corresponding Author

E-mail: duttabob@gmail.com

**Caption**

**Table S1.** Air pollutants and their association with respiratory mortality/morbidity for 18 cities using GAM model during 2000-2020.

**Table S1.** Air pollutants and their association with respiratory mortality/morbidity for 18 cities using GAM model during 2000-2020.

| **SL. No.** | **Cities covered** | **Pollutants** | **Morbidity/ Mortality covered** | **Authors** |
| --- | --- | --- | --- | --- |
|  | **China** |  |  |  |
| 1 | Lanzhou**^1^**,  Jinan**^2^**  Zhoushan**^3^**,  Dongguan**^4^**,  Shijiazhuang**^5^**, Chongqing**^6^**,  Shenzhen**^7^**,  Shanghai**^8^**,  Beijing**^9,13^**,  Guangdong**^10^**, Guangzhou**^11^**,  Hefei**^12^**,  Nanjing**^14^**. | **^1^** SO_2_, PM_10_, NO_2_,  **^2^** PM_2.5_, SO_2_, NO,  **^3^** PM_10_, PM_2.5_, SO_2_, NO_2_ and O_3_,  **^4^** PM_C_, PM_2.5_, SO_2_, NO_2_, O_3_,  **^5^** PM_2.5_, PM_10_, SO_2_, NO_2_ and O_3_,  **^6^** PM_10_, SO_2_, NO_2_,  **^7^** PM_10_, PM_2.5_, SO_2_, NO_2_ and CO, O_3_,  **^8^** PM_10‐2.5_, SO_2_, NO_2_, and O_3_,  **^9^** PM_10_, SO_2_, and NO_2_,  **^10^** NO_2_, PM_10_ SO_2_,  **^11^** PM_10_, PM_2.5_, SO_2_, NO_2_, CO,  **^12^** PM_2.5_, PM_10_, NO_2_, SO_2_, CO,  **^13^** PM_10_, PM_2.5_, PM coarse, NO_2_, SO_2_, CO, O_3_,  **^14^** PM_10_, PM_2.5_, NO_2_, SO_2_, O_3_, and CO. | **^1^**Pulmonary diseases.  **^2^**Pneumonia, asthma, and other pulmonary diseases.  **^3^**Respiratory illness, dermatology illness and ophthalmic illness.  **^4^**Respiratory diseases, COPD, asthma, pneumonia.  **^5^**Respiratory diseases.  **^6^**Respiratory diseases.  **^7^**Pulmonary diseases.  **^8^**Non‐accidental causes, cardiovascular diseases, respiratory diseases.  **^9^**Cardiovascular and respiratory disease.  **^10^**Respiratory diseases, cardiovascular, digestive, urogenital.  **^11^**Respiratory, Cardiovascular.  **^12^**Upper respiratory tract infections.  **^13^**Chronic obstructive pulmonary disease COPD.  **^14^**Childhood lower respiratory diseases (CLRD). | **^1^**Tao et al., 2014; **^2^**Liu et al., 2016; **^3^**Zhang et al., 2018; **^4^**Zhao et al., 2017; **^5^**Song et al., 2018; **^6^**Xia et al., 2019; **^7^**Cai et al., 2019; **^8^**Ge et al., 2011; **^9^**Zhang et al., 2015; **^10^**Zhang et al., 2019; **^11^**Zhang et al., 2014; **^12^**Li et al., 2018; **^13^**Liang et al., 2019; **^14^**Zhu et al., 2017. |
|  | **India** |  |  |  |
| 2 | Delhi**^1^**, Chennai**^2^**, Ludhiana **^3^** | **^1^** PM_2.5_, PM_10_, NO_2_, CO and O_3_,  **^2^** PM_10_, NO_2_, SO_2_,  **^3^** RSPM, NO_x_, and SO_2_. | **^1^** Pulmonary tuberculosis and COPD.  **^2^** All-cause mortality including respiratory.  **^3^** Mortality excluding accidents. | **^1^**Goyal and Kumarb, 2016; **^2^**Balakrishnan et al., 2013; **^3^**Kumar et al., 2010. |
|  | **Iran** |  |  |  |
| 3 | Tehran**^1,2^** | **^1^** CO, PM_10_, PM_2.5_, NO_2_, O_3_, and SO_2_,  **^2^** O_3_, CO, NO_2_, SO_2_, PM_10_ and PM_2.5_, | **^1^** Respiratory and cardiovascular.  **^2^** Respiratory deaths/ mortality from respiratory diseases. | **^1^**Shahi et al., 2014; **^2^**Dehghan et al., 2018 |
|  | **Brazil** |  |  |  |
| 4 | Rio de Janeiro | PM_2.5_ | Pneumonia, acute bronchitis and bronchiolitis, asthma. | Nascimento et al., 2016 |
|  | **Denmark** |  |  |  |
| 5 | Copenhagen | NO_x_, NO_2_, PM_10_ and PM_2.5_, UFPs | Asthma (J45-J46), ICD-10. | Iskandar et al., 2012 |
|  | **Kuwait** |  |  |  |
| 6 | Kuwait | Visibility | Non-accidental mortality. | Achilleos et al., 2019 |

**References**

Achilleos, S., Al-Ozairi, E., Alahmad, B., Garshick, E., Neophytou, A.M., Bouhamra, W., Yassin, M.F., Koutrakis, P., 2019. Acute effects of air pollution on mortality: A 17-year analysis in Kuwait. Environ. Int. 126:476-483. doi: 10.1016/j.envint.2019.01.072.

Balakrishnan, K., Ganguli, B., Ghosh, S., Sambandam, S., Roy, S., Chatterjee, A. 2013. A spatially disaggregated time-series analysis of the short-term effects of particulate matter exposure on mortality in Chennai, India. Air Qual. Atmos. Health. 6: 111e121. doi: 10.1007/s11869-011-0151-6.

Cai, J., Peng, C., Yu, S., Pei, Y., Liu, N., Wu, Y., Fu, Y., Cheng, J. 2019. Association between PM_2.5_ Exposure and All-Cause, Non-Accidental, Accidental, Different Respiratory Diseases, Sex and Age Mortality in Shenzhen, China. Int. J. Environ. Res. Public Health. 16: 401. https://doi.org/10.3390/ijerph16030401.

Dehghan, A., Khanjani, N., Bahrampour, A., Goudarzi, G., Yunesian, M. 2018. The relation between air pollution and respiratory deaths in Tehran, Iran- using generalized additive models. BMC Pulm. Med. 18(1): 49. doi: 10.1186/s12890-018-0613-9.

Ge, W., Chen, R., Song, W., Kan, H. 2011. Daily visibility and hospital admission in Shanghai, China. Biomed. Environ. Sci. 24:117-121. doi:10.3967/0895‐3988.2011.02.005.

Goyal, P., Kumarb, S. 2016. Air Pollution and Respiratory Health in Megacity Delhi, India. J. Adv. Res. Alt. Energ. Env. Eco. 3(3&4): 1-13.

Iskandar, A., Andersen, Z.J., Bønnelykke, K., Ellermann, T., Andersen, K.K., Bisgaard, H. 2012. Coarse and fine particles but not ultrafine particles in urban air trigger hospital admission for asthma in children. Thorax. 67(3):252-257. doi: 10.1136/thoraxjnl-2011-200324.

Kim, H., Kim, W., Choi, J.E., Kim, C., Sohn, J. 2018. Short-term Effect of Ambient Air Pollution on Emergency Department Visits for Diabetic Coma in Seoul, Korea. J. Prev. Med. Public Health. 51(6):265-274. doi: 10.3961/jpmph.18.153.

Kumar, R., Sharma, S.K., Thakur, J.S., Lakshmi, P.V., Sharma, M.K., Singh, T. 2010. Association of air pollution and mortality in the Ludhiana city of India: a time-series study. Indian J. Public Health. 54(2):98-103. doi: 10.4103/0019-557x.73278.

Li, Y.R., Xiao, C.C., Li, J., Tang, J., Geng, X.Y., Cui,L.J., Zhai, J.X. 2018. Association between air pollution and upper respiratory tract infection in hospital outpatients aged 0-14 years in Hefei, China: a time series study. Public Health. 156:92-100. doi: 10.1016/j.puhe.2017.12.006.

Liang, L., Cai, Y., Barratt, B., Lyu, B., Chan, Q., Hansell, A.L., Xie, W., Zhang, D., Kelly, F.J., Tong, Z. 2019. Associations between daily air quality and hospitalizations for acute exacerbation of chronic obstructive pulmonary disease in Beijing, 2013–17: an ecological analysis, Lancet Planet Health. 3: 270–279. doi: https://doi.org/10.1016/S2542-5196(19)30085-3.

Liu, P., Wang, X., Fan, J., Xiao, W., Wang, Y. 2016. Effects of Air Pollution on Hospital Emergency Room Visits for Respiratory Diseases: Urban-Suburban Differences in Eastern China. Int. J. Environ. Res. Public Health. 13(3): 341. doi: 10.3390/ijerph13030341.

Nascimento, L.F., Vieira, L.C., Mantovani, K.C., Moreira, D.S. 2016. Air pollution and respiratory diseases: Ecological time series. Sao Paulo Med. J. 134(4): 315–321. doi: 10.1590/1516-3180.2015.0237250216.

Shahi, A.M., Omraninava, A., Goli, M., Soheilarezoomand, H.R., Mirzaei, N. 2014. The Effects of Air Pollution on Cardiovascular and Respiratory Causes of Emergency Admission. Emergency. 2(3):107-114.

Soleimani, Z., Darvishi Boloorani, A., Khalifeh, R., Griffin, D.W., Mesdaghinia, A. 2019. Short-term effects of ambient air pollution and cardiovascular events in Shiraz, Iran, 2009 to 2015. Environ. Sci. Pollut. Res. Int. 26(7):6359-6367. doi: 10.1007/s11356-018-3952-4.

Song, J., Lu, M., Zheng, L., Liu, Y., Xu, P., Li, Y., Xu, D., Wu, W. 2018. Acute effects of ambient air pollution on outpatient children with respiratory diseases in Shijiazhuang, China. BMC Pulm. Med. 18(1):150. https://doi.org/10.1186/s12890-018-0716-3.

Tao, Y., Mi, S., Zhou, S., Wang, S., Xie, X. 2014. Air pollution and hospital admissions for respiratory diseases in Lanzhou, China. Environ. Pollut. 185: 196-201. doi: 10.1016/j.envpol.2013.10.035.

Vidale, S., Bonanomi, A., Guidotti, M., Arnaboldi, M., Sterzi, R. 2010. Air pollution positively correlates with daily stroke admission and in hospital mortality: A study in the urban area of Como, Italy. Neurol. Sci. 31(2):179–182. doi: 10.1007/s10072-009-0206-8.

Vidale, S., Arnaboldi, M., Bosio, V., Corrado, G., Guidotti, M., Sterzi, R., Campana, C. 2017. Short-term air pollution exposure and cardiovascular events: A 10-year study in the urban area of Como, Italy. Int J Cardiol. 248:389-393. doi: 10.1016/j.ijcard.2017.06.037.

Xia, C., Ma, J., Wang, J., Huang, J., Shen, Q., Chen, Y., Jiang, Y. 2019. Quantification of the Exposure–Lag–Response Association Between Air Pollution and Respiratory Disease Morbidity in Chongqing City, China. Environ. Model. Assess. 24: 331-339. doi: 10.1007/s10666-018-9625-3.

Zhang, Y.L., Zhang, H., Yi, J.P., Zhang, J.J., Dai, X.R., Hang, X. 2018. Effect of Air Pollution on Hospital Admissions of Respiratory, Dermatological, Ophthalmic Diseases in a Coastal City, China. Glob. Environ. Health Saf. 2(1-2): 1-8.

Zhang, Y., Wang, S.G., Ma, Y.X., Shang, K.Z., Cheng, Y.F., Li, X., Ning, G.C., Zhao, W.J., Li, N.R. 2015. Association between Ambient Air Pollution and Hospital Emergency Admissions for Respiratory and Cardiovascular Diseases in Beijing: a Time Series Study. Biomed. Environ. Sci. 28(5):352-63. doi: 10.3967/bes2015.049.

Zhang, Z., Wang, J., Chen, L., Chen, X., Sun, G., Zhong, N., Kan, H., Lu, W. 2014. Impact of haze and air pollution-related hazards on hospital admissions in Guangzhou, China. Environ. Sci. Pollut. Res. 21(6):4236-4244. doi: 10.1007/s11356-013-2374-6.

Zhang, Z., Wang, J., Liu, F., Yuan, L., Yuan, J., Chen, L., Zhong, N., Lu, W. 2019. Impacts of event-specific air quality improvements on total hospital admissions and reduced systemic inflammation in COPD patients. PLoS One. 14(3): e0208687. doi: 10.1371/journal.pone.0208687.

Zhao, Y., Wang, S., Lang, L., Huang, C., Ma, W., Lin, H. 2017. Ambient fine and coarse particulate matter pollution and respiratory morbidity in Dongguan, China. Environ. Pollut. (Barking, Essex: 1987). 222: 126–131. http://dx.doi.org/10.1016/j.envpol.2016.12.070.

Zhu, L., Ge, X., Chen, Y., Zeng, X., Pan, W., Zhang, X., Ben, S., Yuan, Q., Xin, J., Shao, W., Ge, Y., Wu, D., Han, Z., Zhang, Z., Chu, H., Wang, M. 2017. Short-term effects of ambient air pollution and childhood lower respiratory diseases. Sci. Rep. 7: 4414. doi:10.1038/s41598-017-04310-7.
