# Supplementary material for: Air pollution in Delhi, India: It’s status and association with respiratory diseases
Source: PLoS One. 2022 Sep 20;17(9):e0274444. doi: 10.1371/journal.pone.0274444 (PMC9488831; doi:10.1371/journal.pone.0274444)
Supplement: S2 Table — (DOCX) [file pone.0274444.s002.docx]

**Air pollution in Delhi, India:** **Its status and association with respiratory diseases**

Abhishek Dutta ^1, *^, Wanida Jinsart ^1^

^a^ Department of Environmental Science, Faculty of Science, Chulalongkorn University, 254 Phayathai Road, Pathumwan, Bangkok 10330, Thailand

^*^ Corresponding Author

E-mail: duttabob@gmail.com

**Caption**

**Table S2.** Monitoring stations and their geographic coordinates, Delhi.

**Table S2.** Monitoring stations and their geographic coordinates, Delhi.

| **Sl. No.** | **Monitoring stations** | **Station** | **Code** | **Latitude** | **Longitude** |
| --- | --- | --- | --- | --- | --- |
| 1 | CRRI Mathura Road | IMD | 1 | 28.55275 | 77.27341 |
| 2 | ITO, Delhi | CPCB | 2 | 28.63034 | 77.24102 |
| 3 | Jawaharlal Nehru Stadium | DPCC | 3 | 28.5843 | 77.23682 |
| 4 | Lodhi road | IMD | 4 | 28.59024 | 77.21705 |
| 5 | Major Dhyan Chand National stadium | DPCC | 5 | 28.61277 | 77.2363 |
| 6 | Mandir Marg | DPCC | 6 | 28.63952 | 77.20423 |
| 7 | Nehru Nagar | DPCC | 7 | 28.56611 | 77.25106 |
| 8 | Okhla Phase 2 | DPCC | 8 | 28.52968 | 77.27213 |
| 9 | RK Puram | DPCC | 9 | 28.5502 | 77.18518 |
| 10 | Siri Fort | CPCB | 10 | 28.55402 | 77.22764 |
| 11 | Sri Aurobindo Marg | DPCC | 11 | 28.54313 | 77.2007 |
